# Diffusion tensor imaging can detect the early stages of cartilage damage: a comparison study

**DOI:** 10.1186/s12891-015-0499-0

**Published:** 2015-02-21

**Authors:** Taku Ukai, Masato Sato, Tomohiro Yamashita, Yutaka Imai, Genya Mitani, Tomonori Takagaki, Kenji Serigano, Joji Mochida

**Affiliations:** Department of Orthopaedic Surgery, Surgical Science, Tokai University School of Medicine, 143 Shimokasuya, Isehara, Kanagawa 259-1193 Japan; Department of Radiology, Specialized Clinical Science, Tokai University School of Medicine, 143 Shimokasuya, Isehara, Kanagawa 259-1193 Japan

**Keywords:** Magnetic resonance imaging, Diffusion tensor imaging, Apparent diffusion coefficient, Fractional anisotropy, T2 mapping, Cartilage, Osteoarthritis, Knee joint

## Abstract

**Background:**

In the present study, we measured damaged areas of cartilage with diffusion tensor (DT) imaging and T2 mapping, and investigated the extent to which cartilage damage could be determined using these techniques.

**Methods:**

Forty-one patients underwent arthroscopic knee surgery for osteoarthritis of the knee, a meniscus injury, or an anterior cruciate ligament injury. Preoperative magnetic resonance imaging of the knee was performed, including T2 mapping and diffusion tensor imaging. The presence of cartilage injury involving the medial and lateral femoral condyles and tibia plateau was assessed during surgery using the Outerbridge scale. The ADC, T2 values and fractional anisotropy of areas of cartilage injury were then retrospectively analysed.

**Results:**

The ADC results identified significant differences between Outerbridge grades 0 and 2 (*P* = 0.041); 0 and 3 (*P* < 0.001); 1 and 2 (*P* = 0.045); 1 and 3 (*P* < 0.001); and 2 and 3 (*P* = 0.028). The FA results identified significant differences between grades 0 and 1 (*P* < 0.001); 0 and 2 (*P* < 0.001); and 0 and 3 (*P* < 0.001). T2 mapping identified significant differences between Outerbridge grades 0 and 2 (*P* = 0.032); 0 and 3 (*P* < 0.001); 1 and 3 (*P* < 0.001); and 2 and 3 (*P* < 0.001). Both the T2 mapping (R^2^ = 0.7883) and the ADC (R^2^ = 0.9184) correlated significantly with the Outerbridge grade. The FA (R^2^ = 0.6616) correlated slightly with the Outerbridge grade.

**Conclusions:**

T2 mapping can be useful for detecting moderate or severe cartilage damage, and the ADC can be used to detect early stage cartilage damage. The FA can also distinguish normal from damaged cartilage.

## Background

Osteoarthritis (OA) is caused by a range of factors, including age, genetic factors, mechanical stress, and cytokines. OA causes pain, range of motion limitations, and functional joint impairment. A particular characteristic of OA is a reduction in the amount of hyaline cartilage. Proteoglycan depletion and increased cartilage water content are observed initially in OA, and these changes are followed by decreased type II collagen and collagen fiber degradation [1]. As X-ray imaging is not an objective evaluation index based on articular cartilage, it is difficult to evaluate early cartilage damage and repair [2]. Early diagnosis and treatment are important because cartilage contains few cellular components and has a low self-restoration capacity.

In recent years, magnetic resonance imaging (MRI) has become useful for evaluating cartilage damage. Because it depicts cartilage tissue more clearly than X-ray imaging, MRI is used to facilitate multiplanar evaluation. Although standard MRI is relatively insensitive for detection of early cartilage injury, recent advances have improved its ability to evaluate biomechanical and biochemical elements in articular cartilage, including glycosaminoglycan, collagen, and water content. Various types of MRI systems are used for the noninvasive evaluation of cartilage damage, including delayed gadolinium-enhanced MRI of cartilage, T1ρ relaxation time, and ^23^Na spectroscopic imaging. These systems measure proteoglycan depletion, and T2 mapping has the potential for evaluating cartilage water content and collagen fiber direction [3]. Increases in T2 with increased cartilage matrix damage, decreased collagen content, and increased water content have also been reported [3].

Diffusion tensor (DT) imaging is used to detect the water molecule diffusion process [4,5] and is now used to evaluate spinal cord injury [6] and cerebral infarction [7]. DT imaging has also recently been used in evaluation of cartilage damage [4,5]. The apparent diffusion coefficient (ADC) obtained by DT imaging reflects decreased proteoglycan and water content [5], whereas the fractional anisotropy (FA) reflects changes in collagen fiber alignment [5,8,9].

Few reports have investigated whether the Outerbridge grade of the cartilage damage can be assessed using MRI techniques. We graded cartilage damage assessed by arthroscopy according to the Outerbridge classification [10] (Table 1) and measured each grade of cartilage damage using DT imaging and T2 mapping. We also investigated the extent to which cartilage damage could be determined using the Outerbridge grade.

**Table 1:** **Outerbridge classification**

**Grade**	**Property**
0	Normal
1	Cartilage with softening and swelling
2	A partial-thickness defect with fissures on the surface that do not reach subchondral bone or exceed 1.5 cm in diameter
3	Fissuring to the level of subchondral bone in an area with a diameter more than 1.5 cm
4	Exposed subchondral bone

## Methods

### Population

Forty-one patients (14 men, 27 women; 13–78 years old, mean age 51.5 years) who underwent arthroscopic surgery at Tokai University Hospital from April 2010 through May 2011 were included in this study (Table 2). Patients who had undergone arthroscopic surgery were included, whereas patients who underwent total knee arthroplasty and open reduction and internal fixation without arthroscopy were excluded. We also excluded patients who had undergone arthroscopic surgery but had a small area of cartilage damage or mixed grades of cartilage damage, or in whom evaluation with MRI was difficult. The Tokai University Hospital Institutional Review Board for Clinical Research approved the study, and all patients signed the consent form.

**Table 2:** **Indication for operations and arthroscopic findings**

**Indication**	**No. of patients**	**Outerbridge grade**	**No. of patients**
Osteoarthritis	17	0	29
ACL injury	11	1	31
Meniscus injury	7	2	21
Loose body	1	3	20
Osteochondritis dissecans	1		
Osteochondroma	1		
Osteonecrosis	1		
Pigmented villonodular synovitis	1		
Tibia platau fracture	1		

### Image and data analysis

MRI of the knee joint (T2 mapping and DT imaging) was performed on the day prior to the surgery. The sites of the cartilage injury were identified during arthroscopic surgery, and the relationships between the operative findings and the preoperative MRI values in these sites were investigated. When arthroscopic surgery was performed, the knee joint was divided into the following regions: inner femoral condyle, outer femoral condyle, inner tibial condyle, and outer tibial condyle. Three Japanese orthopedic specialists conducted the arthroscopic assessment of the cartilage injuries according to the Outerbridge classification [10] (Table 1).

**Figure 1:**
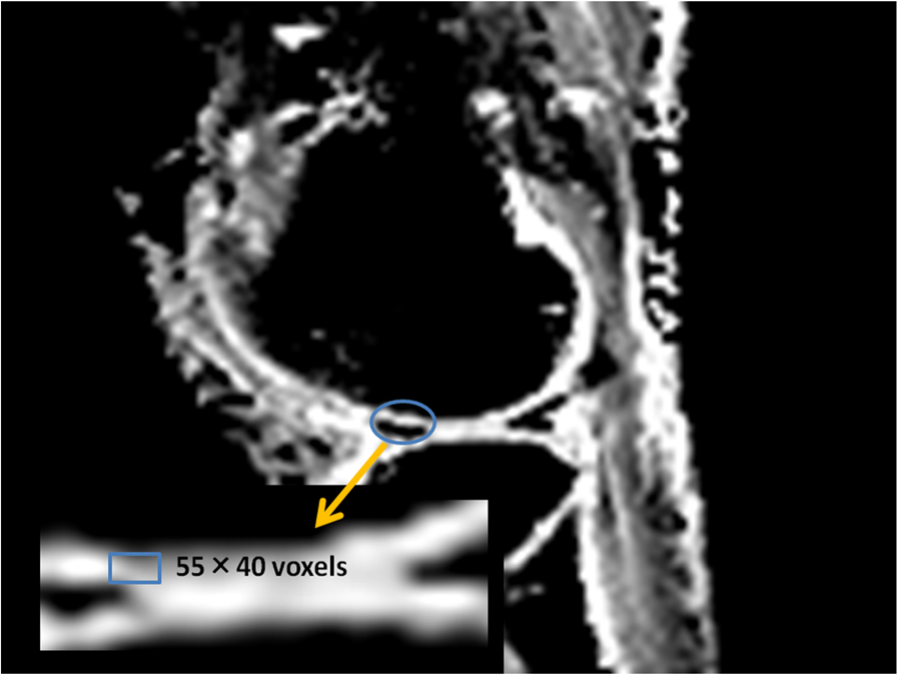
**Region of interest (ROI) settings.** The ROI was set in areas of arthroscopically confirmed cartilage damage, and the ADC, FA, and T2 were measured. The ROI was 55 voxels high and 40 voxels wide, and care was taken to measure all cartilage levels without including the subchondral bone.

The ROIs were measured at all levels, from the cartilage surface to the deep zones, and the subchondral bone was excluded carefully. Each ROI was measured within a range that measured 55 voxels high and 40 voxels wide [5,8] (Figure 1). We evaluated and recorded the Outerbridge grade of the damaged areas of cartilage during surgery, confirmed arthroscopic photographs during measurement of cartilage damage using MRI, and had another doctor reevaluate the Outerbridge grade. Three orthopaedic surgeons and one radiologist, having board certificate of The Japanese Orthopaedic Association and Japan Radiological Society, separately measured the areas of cartilage damage on the MRI. To minimize disparities, the measurements were obtained three times and the mean value calculated.

### T2 mapping

T2 mapping was performed on an Achieva 3.0-T TX scanner (Philips Healthcare, Best, The Netherlands), with the patient’s knees positioned within a TX SENSE Knee eight-channel coil (Philips Healthcare). Imaging was conducted under the following conditions: sequence, multiecho turbo spin-echo (TSE); field of view (FOV), 120 × 120 mm; matrix, 211 × 320; repetition time (TR), 2510 ms; echo time (TE), 16, 32, 48, 64, 80, 96, and 112 ms; turbo factor, 7; slice thickness, 5 mm; gaps, 1 mm; number of excitations (NEX), 1; water fat shift (WFS), 0.882 pixels/429.7 Hz; fat-suppression spectral presaturation with inversion recovery; and scan time, 8 min and 54 s.

### DT imaging

Imaging was conducted under the following conditions: sequence, single-shot, spin-echo echo planar imaging (EPI); FOV, 150 × 150 mm; matrix, 144 × 144; TR, 2200 ms; TE, 68 ms; EPI factor, 73; number of slices, 13; slice thickness, 5 mm; gaps, 1 mm; NEX, 20; WFS, 28.628 pixels/15.2 Hz; fat-suppression spectral attenuated inversion recovery ; MPG, 6; b-value, 600; half-scan factor, 0.678; and scan time, 10 min and 34 s.

### Data processing

From the DT imaging, the six components of the symmetric diffusion tensor were calculated [5]. For each voxel, the three eigenvalues (*λ*1, *λ*2, *λ*3) and their corresponding eigenvectors were calculated. The ADC and FA were calculated from the eigenvalues as follows [8,11,12]:

$$ ADC=\frac{1}{3}\left(\lambda 1+\lambda 2+\lambda 3\right),\;FA=\sqrt{\frac{3}{2}\frac{{\left(\lambda 1-ADC\right)}^2+{\left(\lambda 2-ADC\right)}^2+{\left(\lambda 3-ADC\right)}^2}{\lambda_1^2+{\lambda}_2^2+{\lambda}_3^2}} $$

### Statistical analysis

One-way ANOVA followed by Tukey–Kramer post hoc tests was used to compare the ADC, FA, and T2 between Outerbridge grade. Spearman’s rank correlation was used to identify significant relationships between the Outerbridge grade and the ADC, FA, and T2. *P* values of < 0.05 were considered significant.

## Results

### MRI findings

The ADC for each Outerbridge grade was grade 0, 1.37 ± 0.14 × 10^−3^ mm^2^/s; grade 1, 1.41 ± 0.26 × 10^−3^ mm^2^/s; grade 2, 1.63 ± 0.25 × 10^−3^ mm^2^/s; and grade 3, 1.90 ± 0.40 × 10^−3^ mm^2^/s. The FA for each Outerbridge grade was grade 0, 0.28; grade 1, 0.17; grade 2, 0.14; and grade 3, 0.16. The T2 for each Outerbridge grade was grade 0, 40.1 ± 3.4 ms; grade 1, 41.9 ± 5.5 ms; grade 2, 45.6 ± 7.6 ms; and grade 3, 63.8 ± 5.9 ms.

**Figure 2:**
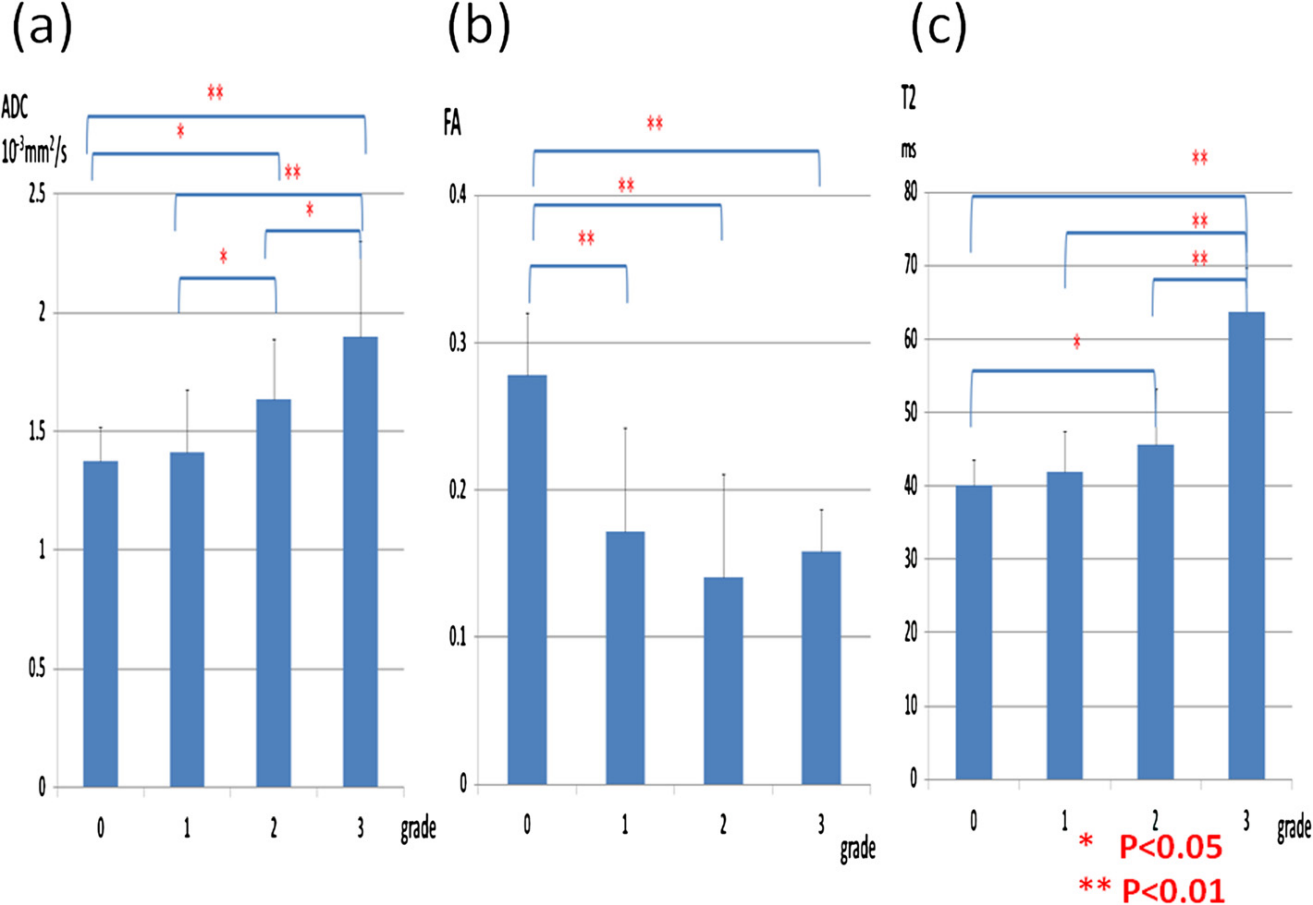
**Comparisons of Outerbridge grade and the ADC, FA, and T2. (a)** Significant differences in the ADC were observed between grades 0 and 2 (*P* = 0.041); 0 and 3 (*P* < 0.001); 1 and 2 (*P* = 0.045); 1 and 3 (*P* < 0.001); and 2 and 3 (*P* = 0.028). No significant difference was observed between grades 0 and 1. **(b)** Significant differences in the FA were observed between grades 0 and 1 (*P* < 0.001); 0 and 2 (*P* < 0.001); and 0 and 3 (*P* < 0.001). **(c)** Significant differences in T2 were observed between grades 0 and 2 (*P* = 0.032); 0 and 3 (*P* < 0.001); 1 and 3 (*P* < 0.001); and 2 and 3 (*P* < 0.001). No significant difference was noted between grades 0 and 1, 1 and 2.

The ADC differed significantly between Outerbridge grades 0 and 2 (*P* = 0.041); 0 and 3 (*P* < 0.001); 1 and 2 (*P* = 0.045); 1 and 3 (*P* < 0.001); and 2 and 3 (*P* = 0.028; Figure 2a). The FA differed significantly between Outerbridge grades 0 and 1 (*P* < 0.001); 0 and 2 (*P* < 0.001); and 0 and 3 (*P* < 0.001; Figure 2b). T2 differed significantly between Outerbridge grades 0 and 2 (*P* = 0.032); 0 and 3 (*P* < 0.001); 1 and 3 (*P* < 0.001); and 2 and 3 (*P* < 0.001; Figure 2c).

### Evaluation of the correlation of Outerbridge grade and MR parameters

**Figure 3:**
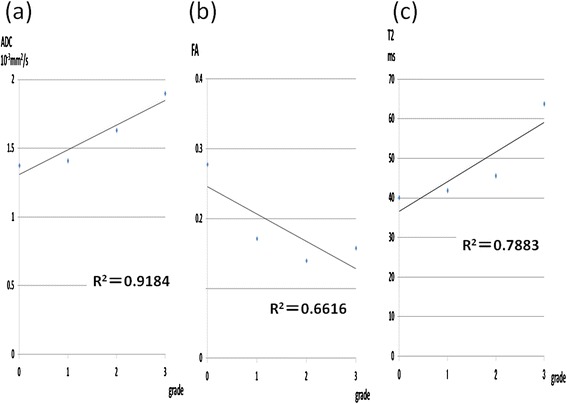
**Relationship between Outerbridge grade and the ADC, FA, and T2. (a)** A significant positive correlation was observed between the Outerbridge grade and the ADC (R^2^ = 0.9184, P < 0.001). **(b)** The FA (R^2^ = 0.6616, P < 0.05) was slightly correlated with the Outerbridge score. **(c)** A significant positive correlation was observed between Outerbridge grade and T2 (R^2^ = 0.7883, P < 0.001).

Significant correlations were observed between the Outerbridge grades and the ADC (R^2^ = 0.9184, P < 0.001; Figure 3a), and between the Outerbridge grades and T2 (R^2^ = 0.7883, P < 0.001; Figure 3c). The FA (R^2^ = 0.6616, P < 0.05; Figure 3b) was correlated slightly with the Outerbridge grades.

### DT imaging of knee articular cartilage

**Figure 4:**
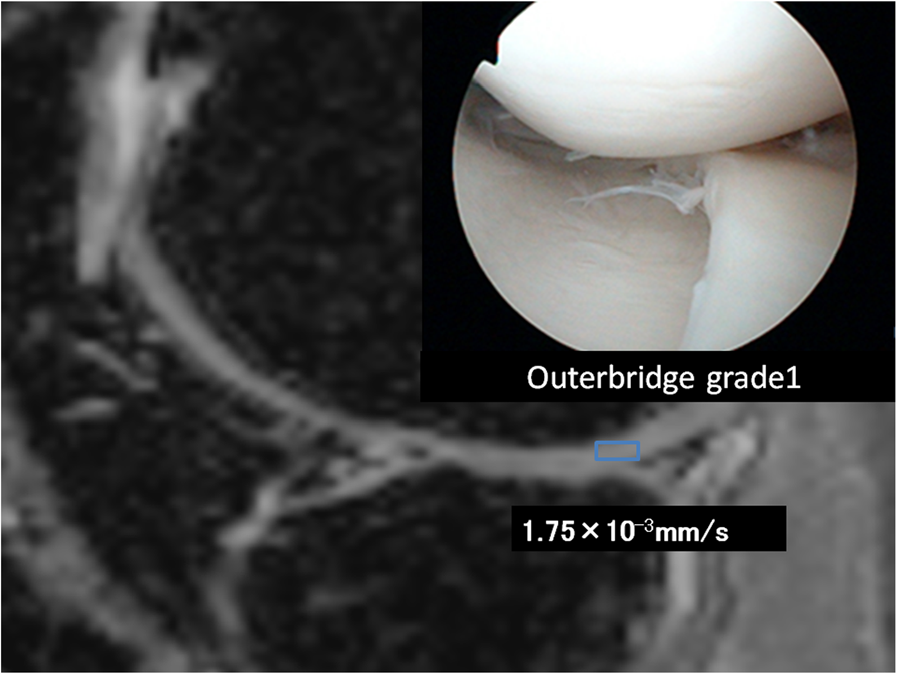
**DT imaging in the early cartilage damage.** The patient underwent surgery for right knee ACL and medial meniscus injury. In the inner femoral condyle, a cartilage crack caused by the medial meniscus rupture was observed to have Outerbridge grade 1 damage. The ADC for this area was measured as 1.75 × 10^−3^ mm^2^/s.

A 21-year-old man sprained his knee while kickboxing. Five months later, he landed awkwardly in a hurdle touchdown and sprained his knee again. Giving way persisted and investigations revealed a right anterior cruciate ligament (ACL) injury and right medial meniscus injury. The patient was treated with ligament reconstruction and meniscal suture (Figure 4).

**Figure 5:**
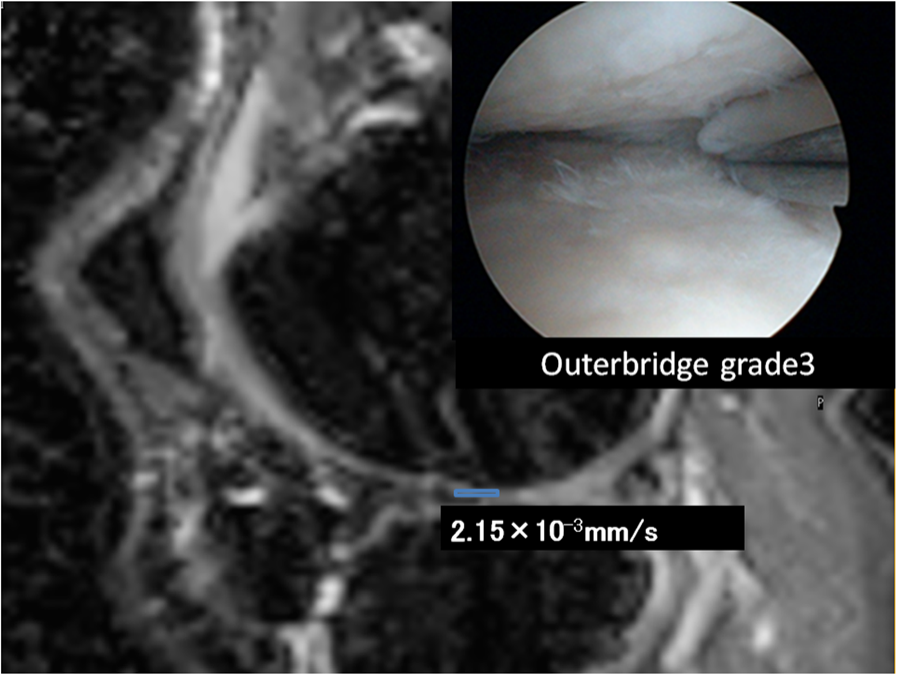
**DT imaging in the severe cartilage damage.** The patient underwent surgery for a right knee medial meniscus injury. Outerbridge grade 3 cartilage damage caused by the medial meniscus rupture was observed throughout the entire inner femoral condyle. The ADC for this area was measured as 2.15 × 10^−3^ mm^2^/s.

A 61-year-old man who had previously undergone meniscectomy in the right knee. He had pain on the inside of the right knee while walking and was being given oral analgesics treatment and injections by his local physician. However, the patient showed no improvement and was referred to our department for further examination. The patient was subsequently diagnosed with osteoarthritis of the right knee and underwent high tibial osteotomy (Figure 5).

## Discussion

T2 mapping in the present study enabled discrimination between Outerbridge grades 0 and 2, 0 and 3, 1 and 3, and 2 and 3, thus allowing detection of moderate or severe cartilage damage. In DT imaging, the ADC enabled discrimination between grades 0 and 2, 0 and 3, 1 and 2, 1 and 3, and 2 and 3 cartilage damage relatively early, and the FA allowed for discrimination between normal cartilage and damaged cartilage between grades 0 and 1, 0 and 2, and 0 and 3.

### T2 mapping

Caution is needed during evaluation because T2 increases considerably with inclinations of about 55° in the direction of the static magnetic field (B0), and a magic angle effect is often observed in the posterior femoral condyle and at the top of the talus [3]. T2 has been reported to reflect the collagen fiber direction and articular cartilage water content. Previous studies that have investigated the use of T2 mapping to evaluate articular cartilage have found the technique to be useful, even for evaluating early stage cartilage damage [13-21]. However, Williams *et al.* [22] found no significant differences between ultrashort echo-time T2 mapping and standard T2 mapping of tissue samples with each grade of cartilage damage.

The present study found significant differences between Outerbridge grades 0 and 2, 0 and 3, 1 and 3, 2 and 3, but no significant differences between grades 0 and 1, and 1 and 2. Threfore, although T2 mapping proved useful for evaluating moderate to severe cartilage damage, it was not useful for evaluating early stage cartilage damage. In T2 mapping, a breakdown of the collagen alignment within cartilage leads to an increase in T2, whereas a decrease in the content of proteoglycans, which form part of the extracellular matrix, has no effect [23,24]. T2 mapping can reflect collagen alignment accurately but may not detect early stage cartilage damage because proteoglycan depletion occurs before collagen depletion in patients with OA-induced cartilage damage [1].

### DT imaging

DT imaging detects water molecule dispersion. In fibrous tissues, water molecule dispersion can occur only in the same direction as the fibers, indicating that the direction of water molecule movement matches fiber alignment. Accordingly, DT imaging can be used to evaluate the direction of articular cartilage collagen fibers and structural anisotropy. Normal articular cartilage exhibits isotropy [12], and cartilage matrix damage leads to anisotropy [22]. Similar to T2 mapping, the results of DT imaging can change according to cartilage depth. The ADC decreases with distance from the surface and toward the subchondral bone, whereas the FA increases closer to the subchondral bone [5]. In DT imaging, the ADC increases with depletion of cartilage proteoglycan and collagen [12,25], both of which are considered to reflect knee articular cartilage degeneration [15,26].

Raya *et al.* [5] collected human articular cartilage and compared the evaluations of the damaged regions between the ADC and actual samples. They found a sensitivity of 95% for cartilage damage detection and an accuracy of 63% for cartilage damage grading. The authors concluded that the ADC is useful for assessing cartilage damage. Meder *et al.* [27] treated cow knee articular cartilage with trypsin before conducting DT imaging and found that the ADC was higher after treatment than before treatment in the trypsin-treated cartilage. The ADC was also reported to increase in human articular cartilage after trypsin treatment [4]. Therefore, the ADC is considered to be useful for evaluating proteoglycan volume.

Few reports have shown that FA is useful for evaluating cartilage damage, and some have stated that the FA does not reflect proteoglycan volume because trypsin treatment barely affects it [4]. Raya *et al.* [26] compared the FA of a normal and an OA knee joint using DTI, reporting that the FA in the OA group declined significantly compared with the normal group, with a sensitivity of 81% and a specificity of 83%. However, in contrast to our case, the evaluation for OA was carried out exclusively by X-*P* in this report, and it is unknown at which grade the evaluation was actually carried out. Moreover, Raya *et al.* [5,26] used 17.6 T and 7.0 T MRI, and they did not report the degree of evaluation that may be carried out with respect to cartilage evaluation upon MRI used in daily clinical practice.

Raya *et al.* [26] measured FA values in an OA group and a normal group, and reported significantly lower values in the OA group. However, no significant difference was observed in grades 1 to 3 of the OA group, and it was difficult to evaluate the cartilage damage solely from FA. According to the report by Deng *et al.* [28], the cartilage is regarded as having weak anisotropy compared with biological tissues, such as the brain and the heart. Consequently, it was believed that although a diagnosis of OA was possible via the investigation into the FA of this study, it was difficult to evaluate the extent of damage caused by OA.

In the ADC, we found significant differences between Outerbridge grades 0 and 2, 0 and 3, 1 and 2, 1 and 3, and 2 and 3. Although no significant difference was observed between grades 0 and 1, the ADC differed from T2 mapping in that it identified a significant difference between grades 1 and 2. Therefore, our results suggest that the ADC is useful for evaluating early stage cartilage damage. A strong correlation was observed between the Outerbridge grade and the ADC, suggesting that the ADC is useful for cartilage evaluation.

## Conclusions

In our study, T2 mapping was useful for assessing moderate to severe cartilage damage (Outerbridge grade 2 and 3), and the ADC was useful for assessing relatively early stage cartilage damage (Outerbridge grades 1 and 2) and severe cartilage damage (Outerbridge grades 2 and 3). The FA can detect cartilage damage but cannot distinguish early cartilage damage from severe cartilage damage.

## Abbreviations

ACL: Anterior cruciate ligament
ADC: Apparent diffusion coefficient
DT: Diffusion tensor
EPI: Echo planar imaging
FA: Fractional anisotropy
FOV: Field of view
MRI: Magnetic resonance imaging
NEX: Number of excitations
OA: Osteoarthritis
ROI: Region of interest
TE: Echo time
TR: Repetition time
TSE: Turbo spin echo
WFS: Water fat shift

## References

[1] Blumenkrantz G, Majumdar S (2007). Quantitative magnetic resonance imaging of articular cartilage in osteoarthritis. Eur Cell Mater.

[2] Eckstein F, Burstein D, Link TM (2006). Quantitative MRI of cartilage and bone: degenerative changes in osteoarthritis. NMR Biomed.

[3] Breuseghem V (2004). Ultrastructural MR imaging techniques of the knee articular cartilage: problems for routine clinical application. Eur Radiol.

[4] Raya JG, Melkus G, Adam-Neumair S, Dietrich O, Mutzel E, Kahr B (2011). Change of diffusion tensor imaging parameters in articular cartilage with progressive proteoglycan extraction. Invest Radiol.

[5] Raya JG, Melkus G, Adam-Neumair S, Dietrich O, Mutzel E, Reiser MF (2013). Diffusion-tensor imaging of human articular cartilage specimens with early signs of cartilage damage. Radiology.

[6] Joseph KB, Harel N, Kim CY, Wang XX, Hasan O, Kauffman A (2013). Diffusion tensor imaging as a predictor of experimental spinal cord injury severity and recovery. Neurosurgery.

[7] Cauley KA, Thangasamy S, Dundamadappa SK (2013). Improved image quality and detection of small cerebral infarctions with diffusion-tensor trace imaging. Am J Roentgenol.

[8] Raya JG, Arnold AP, Weber DL, Filidoro L, Dietrich O, Neumair SA (2011). Ultra-high field diffusion tensor imaging of articular cartilage correlated with histology and scanning electron microscopy. Magn Reson Mater Phy.

[9] de Visser SK, Bowden JC, Wentrup-Byrne E, Rintoul L, Bostrom T, Pope JM (2008). Anisotropy of collagen fibre alignment in bovine cartilage: comparison of polarized light microscopy and spatially resolved diffusion-tensor measurements. Osteoarthritis Cartilage.

[10] Outerbridge RE, Westminster M, Columbia B (1961). The etiology of chondromalacia patellae. J Bone Joint Surg.

[11] Buckwalter JA, Mankin HJ (1997). Articular cartilage II: Degeneration and osteoarthritis, repair, regeneration and transplantation. J Bone Joint Surg Am.

[12] Burstein D, Gray ML, Hartman AL, Gipe R, Foy BD (1993). Diffusion of small solutes in cartilage as measured by nuclear magnetic resonance (NMR) spectroscopy and imaging. J Orthop Res.

[13] Dardzinski BJ, Laor T, Schmithorst VJ, Klosterman L, Graham TB (2002). Mapping T2 relaxation time in the pediatric knee: feasibility with a clinical 1.5-T MR imaging system. Radiology.

[14] Fragonas E, Mlynarik V, Jellus V, Micali F, Piras A, Toffanin R (1998). Correlation between biochemical composition and magnetic resonance appearance of articular cartilage. Osteoarthritis Cartilage.

[15] Frank LR, Wong EC, Luh WM, Ahn JM, Resnick D (1999). Articular cartilage in the knee: mapping of the physiologic parameters at MR imaging with a local gradient coil—preliminary results. Radiology.

[16] Kwee TC, Takahara T, Ochiai R, Katahira K, Cauteren MV, Imai Y (2009). Whole-body diffusion-weighted magnetic resonance imaging. Eur J Radiol.

[17] Mosher TJ, Dardzinski BJ, Smith MB (2000). Human articular cartilage: influence of aging and early symptomatic degeneration on the spatial variation of T2–preliminary findings at 3 T. Radiology.

[18] Nieminen MT, Rieppo J, Toyras J, Hakumaki JM, Silvennoinen J, Hyttinen MM (2001). T2 relaxation reveals spatial collagen architecture in articular cartilage: a comparative quantitative MRI and polarized light microscopic study. Magn Reson Med.

[19] Nieminen MT, Toyras J, Rieppo J, Hakumaki JM, Silvennoinen J, Helminen HJ (2000). Quantitative MR microscopy of enzymatically degraded articular cartilage. Magn Reson Med.

[20] Smith HE, Mosher TJ, Dardzinski BJ, Collins BG, Collins CM, Yang QX (2001). Spatial variation in cartilage T2 of the knee. J Magn Reson Imaging.

[21] Xia Y, Moody JB, Alhadlaq H (2002). Orientational dependence of T2 relaxation in articular cartilage: a microscopic MRI (microMRI) study. Magn Reson Med.

[22] Williams A, Qian Y, Bear D, Chu CR (2010). Assessing degeneration of human articular cartilage with ultra-short echo time (UTE) T2 mapping. Osteoarthritis Cartilage.

[23] Borthakur A, Shapiro EM, Beers J, Kudchodkar S, Keeland JB, Reddy R (2000). Sensitivity of MRI to proteoglycan depletion in cartilage: comparison of sodium and proton MRI. Osteoarthritis Cartilage.

[24] Timothy JM, Bernard JD (2004). Cartilage MRI T2 relaxation time mapping: overview and applications. Semin Musculoskelet Radiol.

[25] Xia Y, Farquhar T, Burton-Wurster N, Vernier-Singer M, Lust G, Helinski L (1995). Self-diffusion monitors degraded cartilage. Arch Biochem Biophys.

[26] Raya JG, Horng A, Dietrich O, Krasnokutsky S, Beltran LS, Storey P (2012). Articular cartilage: in vivo diffusion-tensor imaging. Radiology.

[27] Meder R, Visser SK, Bowden JC, Bostrom T, Pope JM (2006). Diffusion tensor imaging of articular cartilage as a measure of tissue microstructure. Osteoarthritis Cartilage.

[28] Deng X, Farley M, Nieminen MT, Gray M, Burstein D (2007). Diffusion tensor imaging of native and degenerated human articular cartilage. Magn Reson Imaging.

